# A step forward in enhancing the health-promoting properties of whole tomato as a functional food to lower the impact of non-communicable diseases

**DOI:** 10.3389/fnut.2025.1519905

**Published:** 2025-02-05

**Authors:** Pier Giorgio Natali, Mauro Piantelli, Alessandra Sottini, Margherita Eufemi, Cristina Banfi, Luisa Imberti

**Affiliations:** ^1^Mediterranean Task Force for Cancer Control, Rome, Italy; ^2^Department of Medicine and Aging Sciences, Center for Advanced Studies and Technology (CAST), G. D'Annunzio University, Chieti, Italy; ^3^Service Department, Highly Specialized Laboratory, Diagnostic Department, ASST Spedali Civili of Brescia, Brescia, Italy; ^4^Department of Biochemical Science "A. Rossi Fanelli", Faculty of Pharmacy and Medicine, "La Sapienza" University of Rome, Rome, Italy; ^5^Centro Cardiologico Monzino IRCCS, Unit of Functional Proteomics, Metabolomics, and Network Analysis, Milan, Italy; ^6^Section of Microbiology, University of Brescia, P. le Spedali Civili, Brescia, Italy

**Keywords:** antioxidant, functional food, mediterranean diet, non-communicable diseases, tomato

## Abstract

Nutritional interventions facilitating the consumption of natural, affordable, and environment-compatible health-promoting functional foods are a promising strategy for controlling non-communicable diseases. Given that the complex of tomato micronutrients produces healthier outcomes than lycopene, its major antioxidant component, new strategies to improve the health-supporting properties of the berry are ongoing. In this context, a whole tomato food supplement (WTFS), enriched by 2% olive wastewater containing a complex of healthy nutrients with converging biologic activities, has recently been developed, which is superior to those present in tomato commodities or obtained with whole tomato conventional processing methods. WTFS equals the antioxidant activity of N-acetyl-cysteine and interferes with multiple inflammation and cellular transformation-sustaining metabolic pathways. In interventional studies, WTFS inhibits prostate experimental tumors and improves benign prostate hypertrophy-associated symptoms with no associated side-effects. Although WTFS may be susceptible to further improvements and clinical scrutiny, its composition embodies the features of advanced functional foods to ease adherence to dietary patterns, that is, the Mediterranean diet, aimed at contrasting and mitigating the low-grade inflammation, thus being interceptive or preventive of non-communicable diseases.

## 1 Introduction

Due to the increasing incidence of slow-progressing non-communicable diseases (NCDs), which represent the most frequent causes of long-term disability and death worldwide (1), a widening gap between life and healthy life expectancy can be observed (2). This dichotomy is becoming a global health and economic emergency, leading to associated social consequences, especially in low- and middle-income countries (3). Paradoxically, despite NCDs being largely preventable (4), the gap is widening between what we know about their social and biological determinants and what is done for coordinated progressive corrective initiatives. Therefore, the urgent need to reduce or mitigate this alarming trend is emerging through social and individual initiatives. As for the former, current socio-political, economic, and environmental factors are unlikely to improve rapidly. Indeed, they require a coordinated mobilization of societies to reduce the impact of disparities (5), of present conflicts and the outburst of new confrontations (6), the uncontrolled migratory waves (7), and increasing urbanization (8). Regarding personal choices, the well-rooted marketing policies (9), uncensored information released by social media platforms, and supporters of alternative remedies are undermining the ability to make appropriate healthy choices. This erodes the efficacy of health literacy efforts in raising awareness of the risk factors of NCDs (10) and the diminishing healthcare provisions (11). In this uncertain horizon, the compelling issue arises as to what can be done to develop policies aimed at delaying the onset of NCDs and dimming their related disability in a realistic approach beneficial to the fractions of largest populations (12). While acute inflammation can be effectively targeted pharmacologically (13), not infrequently at the cost of severe side-effects, low-grade chronic inflammation (14), which is a shared relevant pathological determinant in NCD incidence and severity (15), remains an unmet therapeutic target. In this challenging endeavor, the lowering of excess production and the increased disposal of free oxygen and nitrogen radicals, the common denominators, and culprits of the pathogenesis of age-related diseases, remains a critical therapeutic goal (16), since it may lead to systemic inflammation (17).

## 2 Dietary nutrients and NCDs

While innovation in drug development for NCDs is advancing at a slow pace, epidemiologic and interventional studies have demonstrated that healthy dietary patterns beyond their nutritional properties may be major players in this regard (18), representing the mainstay of NCD prevention and treatment as well. Thus, advocating the implementation of healthier nutritional recommendations (19), supporting the use of widely available natural resources, is gaining increased attention (20). In particular, the Mediterranean diet (MD) (21), recommending the constant uptake of nutritional antioxidants, has received major attention for being associated with a lower risk of NCDs (22), environmentally sustainable (23), and potentially beneficial also to the population of non-Mediterranean countries (24). MD advocates the consumption of healthy “functional foods” (FF), which despite lacking a conclusive definition at the regulatory level, can be classified from the translational point of view as those “foods containing biologically active natural compounds which, in forms made highly bioavailable, produce healthy effects in addition to nutritional ones, similar to natural food, they can also be consumed as part of a normal diet” (25). Although this definition can be largely agreed upon, the questions of which foods, in which form, how much, and when remain to be answered to optimize their consumption in the real world (26). In this context, the overall well-established healthy biochemical activities exerted by the nutrient complex present in tomatoes are of particular translational relevance.

## 3 Tomato as a major source of dietary antioxidants

The transition to more inclusive global health requires environmentally sustainable policies relying on accessible resources (10): among these, tomatoes are an attractive one (27). As a potent source of natural antioxidants, tomatoes, symbolic fruits of the MD (28), are characterized by overall favorable economic and environmental features (Table 1), largely falling within the frame of the newly proposed paradigm which reconciles different past controversies regarding the health role of food and nutrition (29).

**Table 1:** Tomato’s economic and environmental features.

Economic features	References	Environmental features	References
Worldwide second high-yielding crop	(118)	High biodiversity	(119)
High consumption rate	(120)	High chemodiversity	(30)
Expected 5% increase in the market in the near future	(54)	High nutritional yield	(121)
Unique culinary versatility with wide acceptance in different dietary patterns	(122)	Cultivation requires moderate soil tillage and timely controlled irrigation	(123)
High recyclability of industrial processing waste and packaging	(124)	Growth moderately sensitive to increased environmental CO_2_	(123)
Facilitator of circular economy	(54, 125)	Is considered an “excluder plant” when referred to soil contaminants	(126)
May be the scaffold for the development of a variety of dietary supplements of more targeted health claims	(127)	Organic and conventional cultivations have no significant influence in heavy metal content	(128)
		Residues of pesticides are efficiently removed by washing and cooking	(88, 129)

In this context, epidemiological, experimental, and clinical studies have been primarily focused on lycopene, the red-colored, open-chain beta carotenoid, present in variable concentrations in different tomato cultivars (30), which possesses potent free radicals scavenging properties, enhanced by its ability to induce other endogenous antioxidants (31). Lycopene, present in food in the trans isomeric form (32), undergoes variable individual metabolic rates of transformation (33) into the biologically active cis isomer, acquiring a broad spectrum of healthy biological properties (34), sustaining anti-cancer and anti-inflammatory activity (35). *cis*-Lycopene, which is formed upon variably cooking the fruit (36), has a plasma elimination half-life of 5 days (37) and concentrates in definite anatomical sites (27), representing the preferential biological targets of the carotenoid activity. Due to the limited bioavailability from dietary sources, a constant intake of lycopene-rich foods is needed (38), thus posing a fundamental translational aspect how to exploit its wide beneficial properties.

This gap has been recently overcome by comparative analysis consistently demonstrating that the multi-target healthy properties of tomatoes derive not only from their lycopene content (39, 40). Whole fruits consumption, in fact, by providing a combination of antioxidant and anti-inflammatory nutrients with converging biological activities (41–43), has been proven to result in dose-dependent healthier effects than lycopene supplementation (44–46). This observation is not unexpected since several bioactive compounds endowed with a wide spectrum of biological properties are present in the fruit or are generated following cooking (i.e., by Maillard’s reaction) (47). This knowledge strongly supports the choice of whole tomato as FF for equitable and sustainable diets (45). As recently reported, several clinical studies involving lycopene supplementation and tomato consumption have been performed in humans (48, 49), confirming that tomato consumption and lycopene are both health-supportive (50). However, the statistical strength of these findings is still not verified (51), advocating for the development of new whole tomato standardized formulations containing more lycopene-bioavailable isoforms (52, 53).

### 3.1 Ongoing strategies to improve tomato as FF

Because of its nutritional content, several strategies for improving tomato crop yield and quality are also under exploration, meeting the scaling-up demand for improved commodities (27, 54). Along the same line, the development of friendly technologies using whole tomatoes may represent an advancement in utilizing the fruit as FF. Despite available grounded evidence that heating is the simplest and low-cost processing of the fruit capable of increasing its healthy properties (36, 55, 56), this knowledge has been only recently applied to generate improved tomato food supplements by exploring different controlled heating conditions. Indeed, it is well known that the processing of various raw materials results in a series of by-products containing various substances with a major role in human health and beyond, whose utilization through environmentally friendly technologies can ensure sustainability and added value. This approach originated from early studies demonstrating that a diet enriched (10%) with a whole tomato powder produced by spray drying improved the systemic antioxidant and inflammatory status and prevented the onset of prostate adenocarcinoma in transgenic mice (57) more efficiently than single lycopene diet supplementation (44). To further optimize the tomato powder properties in terms of carotenoid and flavonoid content and to favor the formation of Amadori’s products (47), a new processing treatment of whole fruits has been recently proposed. This included the initial production of a puree by pre-heating at a temperature between 80 and 90°C. This product, not completely freed from seeds and peels, was then concentrated under pressure (300–400 mbar) at lower temperature (60°C). When the refractive index of the concentrate reached 29–30 °Brix, the product was diluted with hot water at a concentration of 12 °Brix and then spray-dried using preferably an inlet temperature of 190°C and an outlet temperature kept below 85°C. The turbine speed was maintained at 2,600 rpm (58). The spray dry method has been selected because it provides high lycopene concentration (27), better preservation of the Amadori’s products (47, 58), long-term storage (59), and a more metabolically effective product (60).

These conditions allowed an optimal recovery of carotenoids, flavonoids, and. in particular, of fructosyl amino acids as a function of the selected time, temperature, and pressure conditions (58).

In view that olives are a source of chemo-preventive nutrients (61), which modulate relevant inflammation-related signaling (i.e., mitogen-activated protein kinases, phosphoinositide 3-kinase, and nuclear factor kappa B subunit 1) (62, 63), 2% of olive waste water was added in the new tomato formulation with the dual aim of protecting the carotenoids from oxidative degradation and of increasing its anti-inflammatory properties. The waste water was obtained by:

- filtrating olive mill waters;
- concentrating the retentate under reduced pressure at a temperature up to 20°C, and to a concentration of 10–15% w/w dry matter;
- spray-drying the concentrated product from step (b) using an inlet temperature of 150–170°C and an outlet temperature below 80°C.

### 3.2 Properties of the new WTFS compared with tomato-based commodities

**Figure 1:**
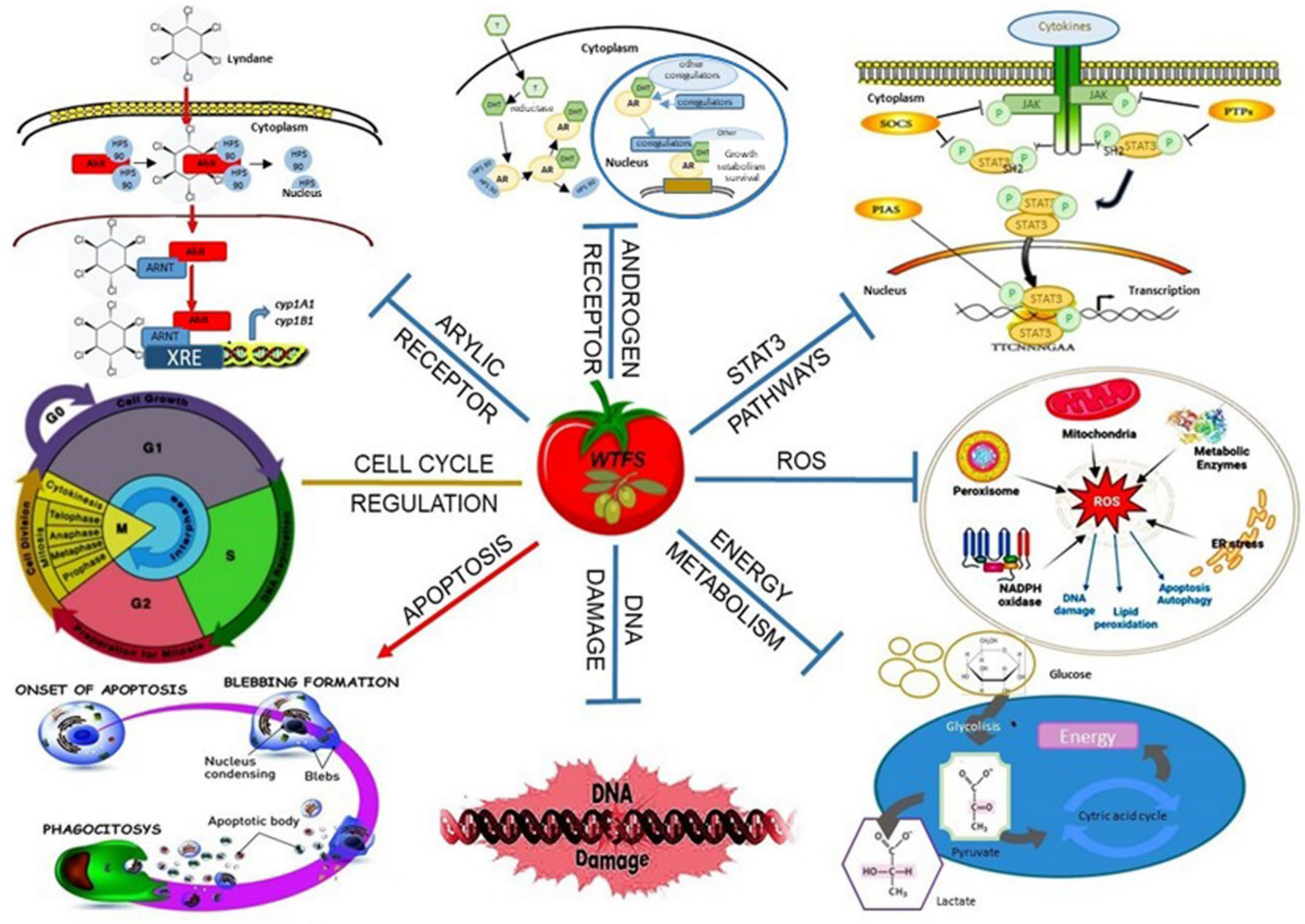
The complex of tomato and olive bioactive compounds in the WTFS is individually or concurrently activating apoptosis, inhibiting DNA damage, the metabolic shift toward the Warburg effect (76), and reactive oxygen species (ROS)-related cell damage (59). The inhibition of STAT-3 oncogene phosphorylation reduces the production of inflammatory cytokines (75). The activation of the androgen and arylic receptors pathways is impaired (66). Overall, the WTFS promotes rebalancing of the cell cycle, protecting cellular homeostasis (66).

WTFS is produced by employing the “Roma” tomato cultivar. These tomatoes were selected because of their high lycopene content (64) and their frequent use in the industrial production of tomato commodities, thus being the appropriate reference to establish whether WTFS indeed represents an improved tomato. The WTFS characteristics include:

- It retains the sensory properties of red tomatoes, thus potentially consumed with good acceptance;
- Further heating for traditional culinary use does not impair its biological activity (65, 66). It may represent an advanced bio-fortifier of a variety of foodstuffs (67), especially in developing countries where supplementation of nutrients-poor diets is increasingly relying on the use of available plants products containing a high nutritional content (68);
- It has an improved nutrient composition compared to the tomato powder generated by heat-processing the fruit through a standard hot break procedure and spray-drying (Table 2) (58). The final product is fully chemical-free and no additive and excipients are present to attain the *in vitro* experimental and human results;
- The concentrations of *cis*-lycopene and other lycopene isomers are higher than those present in tomato consumer products (69) and culinary-treated tomatoes (56, 70, 71). In consideration of their largely variable composition, to reach the daily acceptable requirement of this major antioxidant (0.5 mg /kg body weight) (72), a subject must eat daily exceeding quantities of commercial tomato puree, peeled fruits, or other commodities (69);
- The biological activities of different WTFS production batches are reproducible (66) and the presence of the lycopene *cis*-isomer can contribute to reducing the individual variability in efficiently metabolizing the carotenoid (33), also in view of the powder form (27);
- It contains higher concentrations of flavonoids and newly formed ketosamines, Fru-His compounds, and *β*-carotene, which increases the absorption rate of lycopene (73, 74);
- Its composition is enriched with olive polyphenols, endowed with converging biological activities with tomato nutrients in increasing apoptosis, preventing DNA damage, oxidative stress, receptor modulation, and activation of signal transducer and activator of transcription-3 (STAT-3) a key modulator of the expression of a wide range of oncogenic (66) and inflammation-related pathways (75), and tumor cell energy metabolism (76) (Figure 1);
- The *in vitro* antioxidant activity is comparable to N-acetyl-cysteine (59);
- The power kept sealed for 2 years at constant temperature not exceeding 25°C maintains consistent NAC equivalent antioxidant activities among different batches of WTFS (59, 66) and shows no appreciable differences in biological activities between different production batches (66);
- It has a translational potential in clinical settings. This property has been explored in human benign prostate hypertrophy (BPH), a frequent age-dependent disease sustained by chronic inflammation (77), thus an ideal exploratory target organ (27). The participants of two-phase II prospective (78, 79), randomized double-blinded, placebo-controlled studies were individuals with BPH diagnosed by trans-rectal ultrasound-guided prostate biopsy and/or abnormal digital rectal examination. Of them, 40 were not infected by HIV while 31 were HIV-infected individuals selected among the 3,800 followed in the institution. The treatment with 5 g/day WTFS, which lasted for 2 months, significantly improved the patient’s urinary tract symptoms and quality of life with no associated side effects.

**Table 2:** Nutrient composition (100 g).

Tomato powder		WTFS		
		*Tomato (98%)	*Olive waste water (2%)	
Carbohydrates	66.0 g	63.4 g ± 5.4	Oleuropeinaglycon	5.9 g ± 1.2
Proteins	10.2 g	16.4 g ± 1.7	Ligtrosideaglycon	1.8 g ± 0.8
Lipids	1.6 g	3.4 g ± 0.5	Oleuropein-dialdehydeaglycon	16.2 g ± 1.2
Total carotenoids	142.2 mg	499.5 mg ± 63.1		
All-trans lycopene	109.2 mg	250.8 mg ± 25.1	Ligtroside-dialdehydeaglycon	7.3 g ± 1.0
5-*cis*-lycopene	7.4 mg	34.5 mg ±3.7		
Lycopene isomers	15.7 mg	190.6 mg ± 20.5	Verbascoside	6.4 g ± 1.0
β-Carotene	8.7 mg	22.5 mg ± 2.6	Pinoresinol/deacetoxy-pinoresinol	4.8 g ± 0.9
Lutein	1.2 mg	2.9 mg ± 0.4		
α-tocopherol	1.9 mg	2.3 mg ± 0.3	Thyrosol	2.9 g ± 0.9
Total flavonoids	15.3 mg	199.3 mg ± 51.9	Hydroxy-thyrosol	10.4 g ± 1.1
Quercetin derivates	1.1 mg	140.8 mg ± 31.6	Unindefined polyphenols	8.4 g ± 0.9
Naringenin derivates	4.2 mg	60.8 mg ± 13.2	Polysaccharides	33.6 g ± 1.1
Ketosamines	-	7.5 mg ± 2.4	Humidity	3.5 g ± 0.3
Fru-His	-	0.06 mg ± 0.01		
Fibers	ND	15.8 mg ± 2.9		

The concentration of carotenoids and flavonoids was determined by high-performance liquid chromatography using C30 and C18 chromatographic columns coupled with UV–Vis detection (71). Fru-His was determined by high resolution mass spectrometry using an ExactiveOrbitrap equipment (ThermoFisher, USA). They represent approximately 12% of the water-soluble fraction per dry weight. Concetrations of differenti components are expressed as mean ± SD determined in four WTFS production batches. ND: not determined.

## 4 Potential areas of investigation using WTFS

It is well recognized that tomatoes are a rich source of carotenoids and flavonoid compounds that are capable of reducing platelet aggregation (80, 81) and that water and other forms of fresh tomato extracts can decrease *in vitro, ex vivo*, and *in vivo* platelet activity (82–84). Similarly, being enriched with a complex of anti-platelet aggregating nutrients (85), WTFS induces a dose-dependent inhibition of the STAT-3 transcription factor phosphorylation (66), a relevant player in platelet production and activation (86). Therefore, being WTFS a dietary supplement endowed with anti-platelet activity, it may offer a safe strategy to extend its possible health benefits to cardiovascular health, inflammatory and infectious conditions, and cancer (87).

It is also proven that lycopene protects from several chemical and natural toxins due to the pro-apoptotic properties (88). The evidence that WTFS is capable of blocking *in vitro* the biological effects of lindane (66), a class 1 carcinogen (89), the tomato-dietary supplementation in the form of WTFS or other comparable products (90) opens the possibility of exploring a new remediation strategy to this still globally diffused pollutant. Indeed, despite lindane productionbeing discontinued over 30 years ago, it represents a relevant environmental health risk factor (91) because over 7 million tons remain to be disposed of worldwide (92) and, due to its remarkable stability, at the present, the only remediation is the long-lasting decay. This interventional initiative is likely to be informative in a relatively short time if focused on the young population exposed to lindane. These subjects are known to be affected by impaired spermiogenesis (93), which can be remediated by adherence to the MD (94).

Because any of the common side-effects associated with the culinary use of the fruit have not been so far described for WTFS (78, 79, 95), studies are now ongoing to define its potential efficacy as an alternative to aspirin uptake in individuals with gastrointestinal intolerance. Since lycopene is an inhibitor of endothelial cell stress-induced damage (96), WTFS remains to be explored in aging persons at a higher risk of brain bleeding and in those individuals who may become more vulnerable to bleeding during and after surgery (97). In addition, since WTFS can provide adequate lycopene dietary supply with low-calorie uptake in patients with glucose intolerance, its efficacy may increase insulin sensitivity through inhibition of STAT-3 (98, 99).

Finally, WTFS or similar supplements may currently represent the FF that, facilitating the adherence to the MD, alleviate aging-related carotenoid deficiency (100) and buffer the unhealthy effects of the Western diet (101). A high intake of tomatoes with a low dietary inflammation index (102, 103), enhanced by their cooking processing (104), is highly recommended by MD because tomato consumption is associated with lower overall mortality rates (105). However, MD has some intrinsic translational limitations (106) since its compliance is highly influenced by socio-economic factors (107). In addition, daily consumption of at least three servings of fruits and three of uncooked veggies, recommended by MD as a source of antioxidants, is unattainable in everyday life (94).

## 5 Discussion

According to the Council of Europe guidelines, tomatoes have two health claims: contributing to prostate health and as an antioxidant (108). Although encouraging, the results obtained with WTFS in the treatment of human prostate benign hypertrophy, a heterogeneous group of diseases (109), require further validation, addressing issues such as dosage, scheduling, amenability to combination therapies, and side-effects upon prolonged use. Despite lycopene and tomatoes having been extensively assayed in prostatic cancer prevention and management, these relevant issues have not been fully verified. As WTFS is of reproducible composition (59), it represents a step forward in facilitating the adherence to the otherwise hard-to-follow prostate dietary index (110). In addition, from the translational point of view, the availability of standardized whole tomato formulations of proven superior and well-defined biological activities compared with common source of tomato nutrients and not associated with the common side-effects of culinary tomato consumption will facilitate future clinical studies. The results obtained in the treatment of BPH (78, 79) support this hypothesis and provide informative data to experts of other fields regarding the performance of interventional studies in other areas of interest regarding patient numerosity, dosage, and time of treatment.

The broad biochemical properties of the new supplement (57, 59, 66) may be advantageous in preventing or interfering with the molecular derangements associated with inflammation and malignant transformation fueled by oxidative stress, in tissues where *cis*-lycopene is known to preferentially accumulate (27).

We acknowledge that this review, addressing the “state of the art” in enhancing whole tomato properties as FF in a formulation potentially available to large population fractions may have some limitations. Indeed, information not funneled through no peer-reviewing or in languages other than English may have been missed. Furthermore, comparative analyses between WTFS and thermally treated whole tomatoes obtained in “culinary settings” has been hampered by the lack a detailed composition of the whole fruit preparation obtained (60) or because it generated final products with lower bioavailable antioxidant concentrations (56, 70) than those present in WTFS.

Despite these limitations, advancements in devising friendly, minimal waste-generating technology that improves tomato healthy properties are becoming available. In this endeavor, WTFS, although representing a step ahead, should nevertheless be considered at its inception and seminal to further potential improvements relying on:

- Selection of tomato cultivars with a higher “index of antioxidant nutritional quality” than Roma cultivar (30);
- Devising heating processing, which increases the concentrations of Amadori’s products (56, 74);
- Increasing the olive wastewater content (111);
- Exploring the possibility of developing more focused healthy properties by increasing the concentration of some of its components, that is, lutein (112);

The message often delivered that the regular convivial consumption of tomato is sufficient to benefit from its healthy properties at low cost may be misleading for a fraction of potential consumers. Indeed, the culinary use of tomatoes requires the purchase of fresh or transformed tomato and accompanying food (i. e., legumes, pasta, rice, and meat), the use of condiments (oil and cheese), their cooking, and time required. Furthermore, the dose dependency of tomato-based food consumption benefit (110) implies a continuous monotonous and unhealthy, that is, high-calorie uptake. In this regard, dietary supplementation with improved FF can be of potential help. Because of the present glutting and parceling of the food supplement market, the cost–benefit in consuming new tomato functional food formulations requires wide affordability across economic classes. Although at the present the cost of production of WTFS or similar products for large use is unavailable, a tentative affordable low-cost estimate can be forecast in consideration of the increasing worldwide tomato production, competitive price, spray-drying technology employed for large bulk production, and the commercial availability of olive waste water.

The steady increase in NCD incidence is imposing non-health- and health-related costs on all economies (113). When referring to the latter, healthy diets and nutrition are recognized of primary relevance (114). The development of improved FF of common use may be an attractive choice since they may integrate the dual aim of disease prevention and reduction of severity as well across the homeostasis model (108).

The available data supporting the ability of WTFS to abolish major metabolic pathways generating chronic inflammation offers a large spectrum of *in vitro* and *in vivo* experimentations relevant to settle contentious issues regarding the benefits of lycopene versus whole tomato dietary supplementation.

In view that the benefit of the MD, it can be extendable to populations outside the Mediterranean basin (115). The “Planeterranean” UNESCO project is advocating the use of local food, which may recapitulate the healthy properties of those available in the Mediterranean basin (116), especially in developing countries. In consideration that different cooking habits indeed improve tomato’s healthy properties (117) and that WTFS can undergo further moderate cooking retaining tomato’s sensory properties, it may offer a potential strategy to increase the fruition of the benefits of the MD at a global level by a combined consumption with legumes, tapioca, tuff, and okra, which share nutritional properties with foods available in the Mediterranean area (116).
